# A Reciprocal Link between Oral, Gut Microbiota during Periodontitis: The Potential Role of Probiotics in Reducing Dysbiosis-Induced Inflammation

**DOI:** 10.3390/ijms24021084

**Published:** 2023-01-06

**Authors:** Mattia Di Stefano, Simona Santonocito, Alessandro Polizzi, Rodolfo Mauceri, Giuseppe Troiano, Antonino Lo Giudice, Alessandra Romano, Marco Mascitti, Gaetano Isola

**Affiliations:** 1 Department of General Surgery and Surgical-Medical Specialties, School of Dentistry, University of Catania, 95124 Catania, Italy; 2 Department of Surgical, Oncological and Oral Sciences (Di.Chir.On.S.), University of Palermo, 90127 Palermo, Italy; 3 Department of Clinical and Experimental Medicine, University of Foggia, Via Rovelli 50, 71122 Foggia, Italy; 4 Department of Clinical Specialistic and Dental Sciences, Marche Polytechnic University, Via Tronto 10/A, 60126 Ancona, Italy

**Keywords:** oral microbiome, gut microbiome, periodontitis, periodontal disease, inflammation, probiotics, dysbiosis

## Abstract

Human body is colonized by a florid microbial community of bacteria, archaea, fungi, protists, helminths, and viruses, known as microbiota, which co-evolves with the host and influences its health through all stages of its life. It is well known that oral microorganisms form highly structurally and functionally organized multi-species biofilms and establish a network of complex mutual inter-species interactions having a primary function in synergy, signaling, or antagonism. This ecological model allows the microorganisms to increase their resistance to antimicrobial agents and settle a balanced microbes-host symbiotic relationship that ensures oral and global health status in humans. The host-associated microbiome is an important factor in human health and disease. Therefore, to develop novel diagnostic, therapeutic, and preventive strategies, microbiome’s functions and the reciprocal interactions every microbiome entertains with other microbial communities in the human body are being investigated. This review provides an analysis of the literature about the close connection between the two largest microbial communities in humans: the oral and the gut microbiomes. Furthermore, it focuses on how the alteration of their microbial and functional characteristics can lead to and reciprocally influence the onset of both oral and intestinal microbiome-associated illness, along with the potential role of probiotics in ameliorating inflammation and microbial dysbiosis.

## 1. Introduction

Humans’ digestive tract, oral cavity, skin, airway system, urogenital tract, and others are home to several microbial organisms which have colonized the human body since the dawn of human life and have been vertically transmitted across generations. This host-associated microbial community, including bacteria, archaea, fungi, protists, helminths, and viruses, is known as microbiota, while its collective genome content is known as microbiome, although the two terms are often used interchangeably [1]. Microbiome co-evolves with the host and influences its health through all stages of its life, promoting the development of a mature immune system and the maintenance of systemic homeostasis [2] and inducing host evolution, ranging from microevolutionary to macroevolutionary results [3,4]. The host-associated microbiome is an important factor in human health and disease. Therefore, to develop novel diagnostic, therapeutic, and preventive strategies, microbiome’s functions and the reciprocal interactions every microbiome entertains with other microbial communities in the human body are being investigated. This review provides an analysis of the literature about the close connection between the two largest microbial communities in humans: the oral and the gut microbiomes. Furthermore, it focuses on how the alteration of their microbial and functional characteristics can lead to and reciprocally influence the onset of both oral and intestinal microbiome-associated illness, along with the potential role of probiotics in ameliorating inflammation and microbial dysbiosis.

## 2. Oral Microbiome

**Figure 1:**
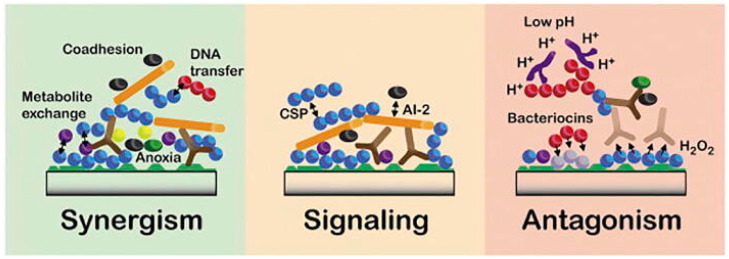
Main interspecies interactions entertained by oral microorganisms for the formation of oral biofilms. Those interactions can be categorized into synergism, signaling, or antagonism. Synergistic interactions are supported by the co-adhesion of microorganisms to other microbes, which enhances multi-species biofilm formation, promotes metabolite exchange, and modifies gene expression through DNA transferring processes, including bacterial conjugation, transformation, and transduction. Signaling interactions are primarily based on Autoinducer-2 (AI-2) and the competence-stimulating peptide (CSP) *quorum sensing* systems, which influence several aspects of microorganisms’ behavior, including their growth, pathogenicity, and autolysis. Antagonistic interactions rely on: bacteriocins, or hydrogen peroxide (H_2_O_2_) production and mechanism lowering microenvironment pH in order to inhibit the growth of specific microbes and gain a competitive advantage. Reproduced with permission. Radaic et al. [22] under Creative Common License.

The human oral microbiome is the second largest, most diverse, and most complex microbial community after the gut one. This unique ecosystem consists of several commensal, symbiotic, and potentially pathogenic microorganisms, including over 700 prokaryotic species, fungi, viruses, and protozoa [5]. Bacteria are the most abundant prokaryotic organisms in the human oral cavity, with approximately 1000 species which can be classified into six main phylotypes of commensal bacteria: *Firmicutes*, *Actinobacteria*, *Proteobacteria*, *Fusobacteria*, *Bacteroidetes,* and *Spirochaetes* [6]. These bacterial microbes are embedded in a self-produced extracellular polymeric matrix (EPM) known as biofilm. It is made up of polysaccharides, proteins, lipids, and extracellular DNA (eDNA) that facilitates the colonization of microorganisms and maintains microbial communities [7,8]. A dynamic process involving the interplay of the host immune system, host genetics, and exposure to both internal and external stimuli leads to the acquisition of the oral microbiome. A crosstalk between maternal microbial antigens and the fetal antigen-presenting cells (APCs) through placental tissues induces a prenatal tolerance to the mother’s microbiome and allows a secure development of a healthy microbiome [9]. Since the infant is exposed to additional possible colonization sources, a later horizontal transmission mechanism, in addition to the maternal vertical transmission mechanism, is crucial in acquiring the physiological microbiome [10,11]. *Streptococcus*, *Staphylococcus*, and *Fusobacterium* species are the most frequently detected early colonizers of the predental oral cavity and remain permanent colonizers into adulthood [12]. More than 100 fungal species have been detected in the oral microbiome of healthy individuals with a prevalence of *Candida* spp. (*C. albicans*, *C. dubliniensis*, *C. rugosa*, *C. pararugosa*) and other fungal species of the *Saccharomycetaceae* family (mainly *Saccharomyces cerevisiae*) [13]. The oral bacterial microbiota and these commensal fungi engage in a variety of synergistic and antagonistic interactions that affect oral bacterial behavior and encourage *C. albicans* to persist in mixed biofilms on natural or synthetic surfaces [14]. Mycobiome pathological alteration occurring in the oral cavity is largely unexplored, and most of the literature is focused on the overgrowth of *C. albicans* in people with a compromised function of the immune system and diabetes mellitus [15,16,17]. Viruses, on the contrary, seem to populate the oral cavity exclusively during pathological conditions, such as Acquired Immuno-Deficiency Syndrome (AIDS) [18,19]. It is well known that oral microorganisms form highly structurally and functionally organized multi-species biofilms and establish a network of complex mutual inter-species interactions having a primary function in synergy, signaling, or antagonism [20]. This ecological model allows the microorganisms to increase their resistance to antimicrobial agents [21] and settle a balanced microbes-host symbiotic relationship that ensures oral and global health status in humans. Certain species of commensal oral bacteria operate as the primary line of defense against the colonization of exogenous pathogens by preventing pathogen adherence and producing bactericidal chemicals such as bacteriocins and hydrogen peroxide (hydrogen peroxide (H_2_O_2_) [22] (Figure 1).

The oral microbial community carries out several beneficial functions, presumably linked to the prevention of colonization by exogenous microorganisms, the stimulation of an appropriate maturation of the immune system, and the maintenance of cardiovascular health [23,24,25,26]. For instance, some commensal oral microorganisms of the genus *Streptococcus* have been shown to hamper the tendency of exogenous pathogens to adhere to the oral substrate, preventing their growth and invasion: *Streptococcus mutans* produces antimicrobial peptides known as bacteriocins under the control of quorum-sensing molecules [27], while *Streptococcus cristatus* can prevent, thanks to its surface protein arginine deiminase (ArcA), *Porphyromonas gingivalis* colonization and biofilm formation in single species or together with *Streptococcus gordonii* [28].

The transition from oral health to disease, which occurs in oral dysbiosis, leads to a significant change in the oral microbiota composition and functions. Compared to healthy subjects, people affected by periodontal disease show considerably larger proportions of the red (*Porphyromonas gingivalis*, *Tannerella forsythia*, *Treponema denticola*) and orange complex species (including bacteria like *Fusobacterium nucleatum* and *Prevotella intermedia*) and significantly lower proportions of the purple complex (comprising *Veillonella parvula* and *Actinomyces odontolyticus*) and *Actinomyces* species [29]. Systemic illness may have a relevant impact on the oral microbiome equilibrium. Microbiota modifications are exacerbated in the case of endocrine, metabolic, and autoimmune systemic diseases, such as diabetes mellitus (DM) [30], rheumatoid arthritis (RA) [31], and systemic lupus erythematosus (SLE) [32]. In fact, these illnesses favor the increase in proinflammatory factors, such as interleukin (IL)-17, which enhance the host inflammatory response and the microbial pathogenicity, leading to a decrease in bacterial taxa associated with health and an increase in bacterial taxa associated with destructive periodontal diseases (PD) [33].

Dysmetabolic diseases can determine an exacerbation of periodontal inflammation in people affected by chronic periodontitis (CP). Type 2 Diabetes mellitus (T2DM), for instance, determinates increased synthesis of multiple inflammatory cytokines, contributing to the damage of the periodontal tissues. Such osteo-immuno-inflammatory factors, including several ILs (among these the previously mentioned -17, and -23), interferon-gamma (IFN]-γ), tumor necrosis factor-alfa (TNF-α), as well as bone-related factors (osteoprotegerin [OPG], receptor activator of nuclear factor-kappa [RANK] B and RANK ligand [RANKL]), are upregulated in poorly controlled patients with type 2 DM and CP. This knowledge suggests that an altered glycemic status represents a relevant risk factor for the severity and progression of PD [34].

Moreover, oral bacteria along with some oral fungi, play a key role in carcinomas genesis and progression through different mechanisms, including the suppression of host immunity system activity and the amplification of its inflammatory response, the neoplastic transformation, the inhibition of cells apoptotic function and the synthesis of carcinogen molecules. Several anaerobic oral bacteria like *Fusobacterium nucleatum (F. nucleatum)* and *Porphyromonas gingivalis (P. gingivalis)* are primarily involved in carcinogenesis, while only a few aerobic oral bacterial species, like *Pseudomonas aeruginosa*, seem to be involved in cancerization processes [35].

## 3. Gut Microbiome

The human digestive tract harbors a wide range of symbiotic and commensal microbes, forming the most complex microbial ecosystem ever found in humans: the gut microbiome. It plays a remarkable role in maintaining humans’ homeostasis for their entire lives. Because microbes start to colonize the neonatal digestive system a few minutes after the birth-giving, the gut microbiome is established in early life [36,37].

The gut microbiome exhibits greater complexity and variety in healthy individuals. Each person presents a gut microbiota characterized by a peculiar microbial composition according to the birth date, the type of delivery, and the methods of milk feeding. This makeup remains relatively constant in adulthood, with inter-individual differences depending mainly on various genetic, nutritional, and environmental factors, such as body mass index (BMI) level, and dietary and lifestyle habits [38]. In contrast, as people age, the diversity of the microbiota declines [39]. The gut microbiome bacterial core is dominated by 4 major phyla: *Firmicutes*, *Bacteroidetes*, *Actinobacteria*, and *Proteobacteria* [40]. In neonatal mice, *Proteobacteria* is the dominant phylum: during this stage of life, *Proteobacteria*-specific IgA maintains the relative bacterial population and controls microbiota maturation [41,42]. *Proteobacteria* amount is subsequently suppressed in typical adult microbiome [41]. The emergence of illnesses may be triggered by the proliferation of some *Proteobacteria* species: *Proteobacteria*’s aberrant growth may cause an energy imbalance among the various species and the inhibition of the growth of other species [43]. The predominant fungal genera detected in gut microbiome include commensal and opportunistic pathogens such as *Candida* (spp. C. *albicans, C. tropicalis, C. parapsilosis*, and *C. glabrata*, *C. krusei*, *C. lusitanae*), *Malassezia*, *Saccharomyces*, and *Cladosporium* as well as environmental and dietary fungi like *Penicillium* and *Aspergillus* [44]. *Candida* spp. are the most represented commensal fungi in the gut microbiome and can act like opportunistic pathogens putting pathogenicity and virulence mechanisms in place [45]. *Malassezia* spp. re lipophilic microbes naturally found on human skin and are related to a wide range of conditions including dermatitis [46].

The gut microbiome is involved in relevant physiological functions since it influences the maturation of the mucosal immune system, the development of cell metabolism, including, synthesis of vitamin K and B12, promotes the integrity maintenance of the GI mucosal barrier, and prevents the intestinal invasion by pathobionts [47,48]. A study of metagenomics shotgun-sequencing conducted on bacterial DNA extracted from fecal samples from 37 Chinese long-living people (age 90 or more) living in Dujiangyan (Sichuan Province, China) has reported significant differences in terms of bacterial composition between healthy and unhealthy people: the healthy group has shown a prevalence of *Bacteroidetes* phylum and genus; on the contrary, the unhealthy group was found a less abundance of *Bacteroides* and a higher abundance in *Firmicutes* phylum and *Streptococcus* genus [49]. Mounting evidence suggests that perturbations of the composition and function of the gut microbiome can lead not only to dysmetabolic illness [50] but can also determine extra-intestinal diseases, ranging from immunological and cardiovascular to neurological/neurodegenerative conditions [51,52,53]. The onset of systemic disorders could be driven by the proliferation of opportunistic pathogenic bacteria and the depletion of anti-inflammatory bacteria. Through the toll-like receptor 4, endotoxins produced by gut microbiota can be released into the bloodstream and cause enterocytes to produce pro-inflammatory cytokines [54]. On the other hand, some gut bacteria are known to have a beneficial influence on systemic inflammation: certain strains of *Faecalibacterium*, such as F. *prausnitzii*, produce anti-inflammatory cytokines lowering the production of pro-inflammatory IL-12 and IFN-γ and enhancing the production of anti-inflammatory IL-10 [55,56]. In addition, intestinal bacteria could influence host inflammatory status, including the periodontal tissue inflammation and the consequent destructive outcomes of this illness, through soluble inflammatory pathways, such as the NF-B pathway and the toll-like receptor signaling pathway affected by *Escherichia coli* and *Faecalibacterium prausnitzii* [57,58,59,60].

## 4. Impact of Dysbiosis during Periodontitis

**Figure 2:**
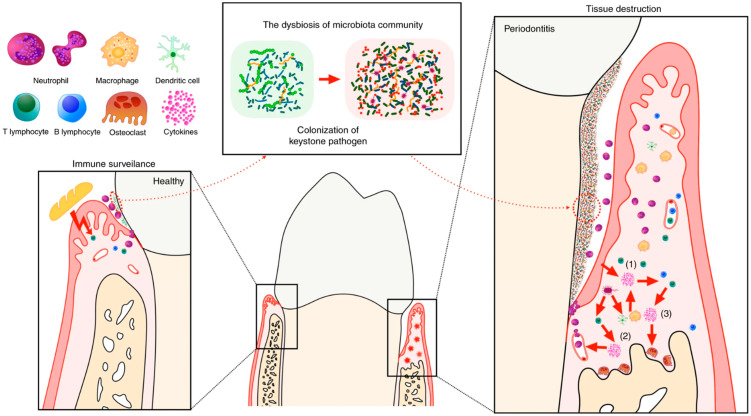
Periodontal tissue in a healthy state and the pathogenesis of chronic periodontitis (CP). In a healthy state, there is a balance between microbial changes and host immune response, with infiltrating neutrophils and resident immune cells in the gingival sulcus, performing immune surveillance. Colonization of periodontal keystone pathogens over-activates the host immune response, leading to the amplification of the proinflammatory cytokine cascade. The first wave of cytokine production is caused by the interaction of the microbiota with all host cells (1), and it primarily contributes to the amplifying of the proinflammatory cytokine cascade and the recruitment, activation, and differentiation of certain immune cells. After being stimulated by the microbiota, mononuclear phagocytes and antigen-presenting cells release a series of cytokines (2) that are intimately linked to the differentiation of a certain subgroup of lymphocytes. Each of these cell subsets secretes a specific pattern of cytokines, which may function as a direct effector or a positive-feedback factor (3) and ultimately result in potential tissue destruction. Reproduced with permission from Pan et al. [69] under Creative Common License.

Dysbiosis consists of an alteration of metabolic activity and/or of quantitative and/or qualitative composition of the microbiota (metabolic dysbiosis and/or taxonomic dysbiosis), generally associated with a decrease in the microbial diversity and the expansion of specific bacterial taxa [61]. Considering the critical role that human microbiota, particularly the gut one, take up in ensuring nutritional, immunological, and developmental benefits, the transition from eubiosis to dysbiosis in human microbial communities can lead to many diseases [62]. The link between dysbiosis and the onset of illness is increasingly evident in the etiopathogeneses of PD [63,64], a broad range of multifactorial inflammatory dental plaque-induced diseases affecting soft and connective teeth-supporting tissues. Gingival inflammation is a necessary precondition for the progression to periodontitis, which is driven by multiple and reinforced interactions between the dysbiotic oral microbiome and a dysregulated inflammation host response in susceptible individuals with various genetic, systemic, and oral factors [65,66]. The dysbiosis of subgingival microbial communities sustains local inflammation: dysbiotic microbes in the local plaque environment trigger host immune responses and facilitate pathobiont microbes’ persistence, with a consequent over-activation of the immune response and long-term inflammation status that promotes gingiva, periodontal ligaments, and alveolar bone irreversible destruction. Although oxygen consumers like *Neisseria* spp. and *Streptococcus* spp. are also a part of the taxa enriched in gingivitis, communities in gingivitis are enriched mostly for gram-negative anaerobic species [67]. In the early stages of periodontitis, the innate immune system’s resident cells (epithelial cells and fibroblasts), phagocytic cells (macrophages and neutrophils), complement proteins, and neuropeptides support a physiological acute inflammation reaction (gingivitis) to supragingival and subgingival plaque. TNF, IL-1, and IL-6, as well as other pro-inflammatory cytokines, are primarily responsible for promoting the migration of cells to infection sites during this process [68]. Damage to the periodontal region is promoted by these pro-inflammatory cytokine clusters (IL-1, IL-6, and TNF families) that can stimulate the expression of the receptor of RANKL crucial for the maturation and activation of osteoclasts and determine the subsequent alveolar bone loss [69,70]. Modifications occurring in oral microbiota composition during the transition between health status and periodontal disease remain today a field of study since different Authors have come to conflicting conclusions, reporting a loss of microbial diversity in some cases and an increasing level of microbial diversity or no significant differences in composition in others [71]. Abusleme L. et al. [67] report that bacterial communities undergo a significant change during periodontitis characterized by the enrichment of mostly gram-negative anaerobic species. An analysis of publicly available 16S ribosomal RNA gene amplicon datasets to define the microbial signatures of health, gingivitis, and periodontitis reports more species detected in disease than in health. The species associated with health seem to be involved in the early phases of biofilm colonization and formation and tolerate higher levels of oxygen in the environment; they include aerobe and facultative anaerobic microorganisms, such as *Corynebacterium*, *Streptocccus* and *Actinomyces* genera. On the other hand, gingivitis has been observed a higher richness and diversity of species than in health and periodontitis, with amount of gram-negative anaerobic species, including facultative anaerobes microorganisms. These results are justified by the fact that as dysbiosis and the inflammation proceeds, certain gram-negative anaerobic species such as *Fusobacterium* spp., especially all *F. nucleatum* subspecies, become prevalent in periodontitis, reducing the global diversity in comparison to gingivitis. Moreover, *F. nucleatum* can lead to a polymicrobial mixed infection in a murine model, co-aggregating, and coinfecting with *P. gingivalis* and inducing a major alveolar bone loss in mouse periodontal tissues and stronger inflammatory response compared with mono-infection models of experimental periodontitis [72]. A study conducted on patients with chronic periodontitis by performing 16S rRNA gene sequencing revealed that at the phylum level, *Actinobacteria*, *Spirochaetes*, *Synergistetes*, and *Tenericutes* are the more abundant bacterial phyla in the salivary sample; these phyla, considering the species level, were assigned to *Eubacterium saphenum*, *Tannerella forsythia*, *Filifactor alocis*, and *Parvimonas micra*, four species previously associated with periodontitis also in plaque samples [69] (Figure 2).

## 5. Mutual Interactions between the Oral and Gut Microbiome

According to the current evidence, the oral and gut microbiota are intimately associated. Studies in mice have demonstrated that oral microbes can ectopically colonize the gastrointestinal (GI) tract. In the context of perturbed gut microbiota, oral microorganisms can contribute, through the stimulation of T-helper-cell (Th1) induction in the gut-associated lymphoid tissue, to the development of digestive systemic diseases linked to severe colonic inflammation, such as colorectal cancer (CRC), irritable bowel syndrome (IBS), and inflammatory bowel disease (IBD) [73,74,75,76].

Several oral bacteria could potentially migrate to the GI tract primarily via hematogenous or enteric routes, inducing an abnormal immune response in the GI tract and leading to intestinal inflammation. Periodontitis results in the proliferation of oral commensal pathobiont-reactive Th17 cells that transmigrate to gut mucosa and exacerbate intestinal inflammation [77]. Key periodontal pathogens such as *F. nucleatum* and *P. gingivalis* can spread into systemic circulation during PD [78] and can also translocate to the gut, overcoming the several physical and/or chemical barriers offered by the GI tract, such as gastric acidity and bile acid and the uncorrupted integrity of intestinal mucosa [79]. *P. gingivalis*, one of the major periodontopathic bacteria, when orally administered to C57BL/6 mice, seems to induce changes in gut microbiota composition. These modifications include: (a) the expansion of unclassified *Muribaculaceae* and *Prevotella* spp.; (b) the downregulation of the expression of the tight junction proteins zonula occludens-1 (ZO-1) and occluding, which leads to the alteration of the physiological barrier function of intestinal mucosa; (c) the increase releasing of proinflammatory cytokines such as IL-6, IL-12, and IFN-γ and the consequent enhancement of inflammatory responses [80]. Analog effects on the expression of tight junction proteins ZO-1 and occluding and the secretion of proinflammatory cytokines have been observed to be promoted by *F. nucleatum* in mice with dextran sulfate sodium (DSS)-induced colitis [81].

It has been shown that the alteration of nitrate-reducing bacterial components of the gut and oral microbiome, can determine the dysfunction of the nitric oxide (NO) signaling system. NO is a small gaseous signaling molecule that plays a key role in the regulation of vascular tone, neurotransmission, mitochondrial respiration, and skeletal muscle contractile function [82]. Nitrate-reducing bacteria include genera comprised in the phyla *Firmicutes* (*Staphylococcus, Streptococcus*, and *Veillonella)* and *Actinobacteria* (*Actinomyces)* and produce nitrate reductases (NaRs), a set of enzymes involved in the reduction in salivary and dietary inorganic nitrate (NO_3_^−^) to nitrite (NO_2_^−^) and then nitric oxide (NO) other reactive nitrogen intermediates into nitrate–nitrite–NO pathway. Considering the relevant role of NO in maintaining physiological integrity in various districts of human body, a decreased availability of NaRs enzymes can alter the nitrate-nitrite-NO pathway and affect availability of bioactive NO, with considerable consequences for individual’s overall health, including the development of human cardiovascular, pulmonary-vascular, neuromuscular, and metabolic diseases, such as arterial hypertension (AH), pulmonary arterial hypertension (PAH), Alzheimer’s disease, etc. [82,83].

The increasingly clear connection between oral health and systemic health is strengthened by the emerging role of the oral-gut-liver axis in the pathogenesis of gastrointestinal and liver diseases. This axis includes the oral cavity and its microbiome with oral pathobionts and potentially pathogenic microbes participating in the reciprocal microbial and immune relationship between the oral cavity and GI tract [79,84]. Recent research has highlighted the potential role of some pathogenic microorganisms such as *P. gingivalis* and *F. nucleatum* to stimulate the comorbidity of PD and IBD, two inflammatory diseases that share microbiological and immunological traits [85,86]. Said diseases have been shown to coexist in people and could share some risk factors, initiating secondary inflammatory infections in other areas of the human body [87]. These findings suggest a connection between gut inflammation and PD established through microbial communication.

Furthermore, the detection of identical strains of *F*. *nucleatum* in colorectal and saliva specimens of patients with CRC indicates *F*. *nucleatum* seems to be related to CRC development and pathogenicity [88]. This role was confirmed by two experimental studies revealing *F. nucleatum* may promote a tumor-immune evasion mechanism in humans, inhibiting T-cell-mediated immune responses against CRC through the interaction of its Fap2 protein with TIGIT receptors expressed by tumor-infiltrating lymphocytes [89,90]. The adhesion of bacterial cells to the intestinal epithelium aids the colonization of the gut by oral pathogens through the upregulation of certain cell adhesion molecules. In experimental animal models, including DSS-induced colitis models, upregulation of mucosal addressing cell adhesion molecule 1 (MadCAM-1), a ligand for α4β7 integrin, a gut-homing molecule expressed in vessels in the colonic lamina propria of patients with IBD, suggests that improved interaction between α4β7 integrin and MadCAM-1 contributes to accelerating the influx of oral Th17 cells into the gut, with consequent aggravation of the intestinal pathology [91]. Subsequently, the integrity of intestinal mucosa is harmed when certain oral pathobionts synthesize and release some cytotoxic substances, such as hydrogen sulfide (H_2_S) and bacterial toxins [92].

Apart from the gut, lungs, heart, placenta, and brain are other distant organs where oral cavity-associated bacteria have been detected [76]. This observation suggests that oral microbes are associated with other common chronic conditions. While the role of oral microorganisms in the development of cardiovascular disease has been analyzed and demonstrated to a sufficient extent [93,94,95,96], more recently the attention of researchers has focused on the connection between oral microbiota and the brain. A review of the literature conducted by Y. Maitre et al. reported a correlation between the oral microbiome and the pathophysiology of some mental health disorders, including Alzheimer’s disease, autism spectrum disorders, Down’s syndrome, and bipolar disorders [97].

## 6. Probiotics

Probiotics are living nonpathogenic bacteria and yeast administered in adequate amounts, as foods or dietary supplements, to provide health benefits to the host [98,99,100]. Several microorganisms have been shown to possess probiotic properties, including *Lactobacillus*, *Bifidobacterium*, *Saccharomyces*, *Enterococcus*, *Streptococcus*, *Pediococcus, Leuconostoc*, *Bacillus*, *Escherichia* genera [101]. The beneficial effects of probiotics were identified in the 19th century by Ilya Metchnikoff [102], who was awarded the 1908 Nobel Prize in Physiology or Medicine for discovering the innate cell-mediated immune response of phagocytosis against microbes during infections [103]. Metchnikoff said: “*The promise of microbiome research results largely on the future of probiotics…. Eventually, it may become possible to restore the health of a depleted microbiome simply by swallowing a capsule crammed with billions of bacterial cells, or by eating yogurt*” [102]. Probiotics and their potential effects have attracted the attention of many researchers over time and are still being investigated to this day.

**Figure 3:**
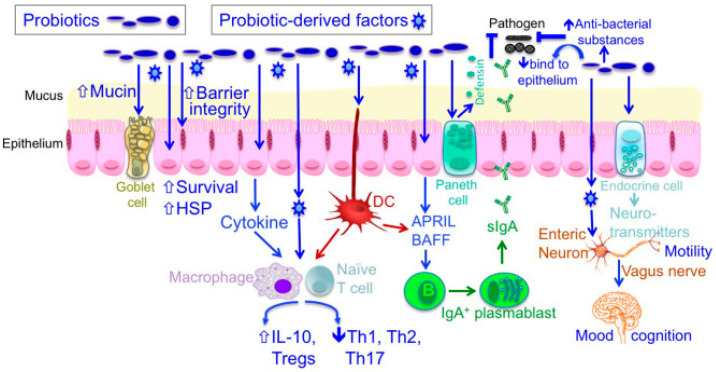
Cellular and molecular probiotics actions. Probiotics guaranteed the preservation of barrier structure and function and the blockade of apoptosis in intestinal epithelial cells through mucus production, survival, and cytoprotective responses. Some probiotic-derived factors, such as polysaccharide A (PSA), modulate the host immune responses through the toll-like receptors (TLR)-2 in dendritic cells (DC), resulting in increased regulatory T cells and interleukin 10 (IL-10) production as well as the downregulation of T helper cells (Th1, Th2, Th17) activity. p40, a Lactobacillus rhamnosus GG (LGG)-derived soluble protein, upregulates EGF-receptor-dependent production of a proliferation-inducing ligand (APRIL) in intestinal epithelial cells, contributing to increased conversion of B cells in plasmablast and consequently enhanced production of secretory immunoglobulin A (sIgA) in the intestinal tissue. Probiotics participate in gut–brain communication through the activation of the vagal pathway—independently of brain-derived neurotrophic factor production—positively affecting responses to stressful, anxiogenic, and depressive stimuli. Reproduced with permission from Yan et al. [105] under Creative Common License.

Up to now, we know that probiotics’ action is based on several cellular and molecular mechanisms involving mainly microbes’ products and metabolites [104]. Various probiotic effector molecules mediating specific probiotics mechanisms of action have been identified. They are related to:

1. *Modulation of microbiota composition*—The inhibition of colonization and invasion by pathogenic microorganisms occurs through the synthesis of antimicrobial metabolites and competitive inhibition of pathogens and toxins for adhering surfaces of the intestinal epithelium [105], for instance, the production of bacteriocin [106,107].
2. *Modulation of the immune cells and immunity*—The host’s innate and adaptive immune responses are influenced by the interaction with the toll-like receptors (TLRs) on the surface of the intestinal epithelial cells or immune cells associated with intestinal mucosa [107]: this molecular interplay results in altered epithelial signaling, leading to the suppression of proinflammatory cytokine production and the enhancement of anti-inflammatory cytokine synthesis (exopolysaccharide, short-chain fatty acids) other than the anti-tumor immunity [108,109].
3. *Enhancement of intestinal barrier function*—The preservation of intestinal epithelium integrity and homeostasis is ensured by restoring epithelial barrier function through the promotion of tight junction functionality [110] and synthesis of mucin and IgA production (protein p40) [111] as well as the genesis of cell survival and protective responses (lipoteichoic acid) [105].
4. *Regulation of the enteric and central nervous systems*—The increased production of neurotransmitters—such as gamma-aminobutyric acid (GABA), N-acetyl aspartate (NAA), and glutamate [112]—and the activation of the vagus nerve (cranial nerve X) as the main anatomical and functional part of the parasympathetic nervous system [105], determinate a modification of endocrine functions and motility and secretion of GI tract.
5. *Cholesterol-lowering action*—Reduction in blood cholesterol is performed via numerous mechanisms, studied in vitro (conversion of cholesterol into coprostanol, incorporation of cholesterol into the cellular membranes, synthesis of new bile acids, etc.) [108,113].
6. *Blood pressure-lowering effect*—thanks to several ways, including reduction in reactive oxygen species production [114,115] and inhibition of angiotensin-converting enzyme [105] (Figure 3).

## 7. Role of Probiotics against Dysbiosis

Probiotics have been shown to re-establish the microbial equilibrium and prevent various diseases of the GI tract e.g., necrotizing enterocolitis, antibiotic-associated diarrhea, relapsing *Clostridioides difficile* colitis, *Helicobacter pylori* infections and IBD [116,117,118]. D. Singh et al. [119] have suggested a potential role of probiotics in the prevention and management of microbiota dysbiosis-associated cancer, in particular liver and oral cancers, for their modulating action on microbiota composition and host immune responses. Microbiota dysbiosis can drive hepatocellular carcinoma (HCC) with several mechanisms, including (a) translocation of gut bacteria and their metabolites to the liver through the portal veins and consequent increasing production of inflammatory mediators responsible for hepatocarcinogenesis; (b) production of bacterial endotoxin Lipopolysaccharides (LPS) and subsequent activation of TLRs promoting the activation of intracellular survival signaling pathways, the synthesis of pro-inflammatory cytokines and mitogen molecules; (c) increased synthesis of deoxycholic acid (DCA) leading to the production of tumor-promoting factors and pro-inflammatory cytokines as well as reactive oxygen species (ROS) and reactive nitrogen species (RNS) inducing DNA damage. Analog mechanisms have been illustrated for the oral squamous cell carcinoma (OSCC) and consisting of: (a) release of pro-inflammatory cytokines—IL-1, IL-6, IL-27, IL-23, TNFα—and matrix metalloproteinases (MMPs) 8, 9 and 13; (b) production of ROS and RNS inducing DNA damage, alteration of epithelial barriers—which predispose the oral mucosa to the development of chronic pre-cancerous lesions—pro-tumoral genetic changes and epigenetic modification (e.g., alteration of onco-miRNA or DNA methylation phenomena). Even though most of the research about probiotic action focuses on gastrointestinal illness treatment and prevention, those microorganisms have been shown to be effective in treating dental issues caused by infections and microbiota imbalances, such as dental caries, and periodontal disease [120]. The several methods of action of probiotics, including the modulation of the oral biofilm composition and indirect effects on the local and systemic immune responses and the mucosal permeability, have been discussed in the previous section. Studies evaluating the effects of probiotics on dental health suggest *Lactobacillus rhamnosus* might be useful in the prevention of dental caries: long-term consumption of milk containing *Lactobacillus rhamnosus* GG, ATCC (LGG) has been demonstrated to reduce the risk of caries in children (1–6 years) and short-term consumption of cheese containing *L. rhamnosus* GG ATCC 53103 and *L. rhamnosus* LC 705 can reduce the counts of *Streptococcus mutans* and salivary yeasts in young adults (18–35 years, mean age 24.5 years) [121,122]. Furthermore, probiotics have been shown to have the potential to alter the sub-gingival microbiota’s composition, significantly reducing the concentration of the main periodontal pathogens [123]. *Lactobacilli* and *Streptococci* strains have been shown to possess antibacterial activity against known periodontal bacteria like *P. gingivalis*, *Prevotella intermedia*, *Aggregatibacter actinomycetemcomitans* and *F. nucleatum* in vitro studies [124], therefore they could be a potential adjuvant in treatments for periodontal disease as also concluded by A. Vives-Soler and E. Chimenos-Küstner after a systematic analysis of the available data [125]. Moreover, a randomized, double-blind, placebo-controlled clinical trial aiming to assess the potential role of orally administered *L. rhamnosus* SP1 as an adjunct to non-surgical therapy of CP, has shown that people in the test group exhibit a notable reduction in PD compared to the ones of the placebo group [126].

## 8. Conclusions

Oral and gut microbiomes can influence physiological functions and pathological events in a mutually dependent way. The translocation of oral pathogenetic microbes to the gut and vice versa can modify the microbial equilibrium in both ecological environments and modulate the pathogenesis of both oral and intestinal diseases. For accurate diagnosis/prognosis and efficient treatment, it will be useful to have a better knowledge of the function of the oral-gut microbiome axis in the regulation of pathogenetic phenomena.

## 9. Future Perspective

Intense research is still being conducted on the microbiological and immunological relationship between the mouth and the gut in the emergence of intestinal inflammation. In this context, additional cohorts and longitudinal investigations are needed to assess the importance of the oral-gut connection during the development of intestinal inflammation and the coevolution of intestinal and periodontal diseases.

## Data Availability

Data are available from the corresponding author upon reasonable request.
